# Lipoxin A4 and Resolvin D1 Preserve Neural Inductive Capacity of Dental Pulp Stem Cells Cultured Under Inflammatory Conditions

**DOI:** 10.1002/cbin.70163

**Published:** 2026-05-19

**Authors:** Ana Paula Turrioni, Geovana Pires da Silva, Yan Xu, Alpdogan Kantarci, Hatice Hasturk, Ricardo A. Battaglino, Leslie R. Morse

**Affiliations:** ^1^ Department of Pediatric Dentistry and Orthodontics, School of Dentistry Federal University of Uberlandia Uberlandia Minas Gerais Brazil; ^2^ ADA Forsyth Institute Somerville Massachusetts USA; ^3^ University of Minnesota Minneapolis Minnesota USA; ^4^ Department of Physical Medicine and Rehabilitation, Miller School of Medicine University of Miami Coral Gables Florida USA

**Keywords:** inflammation, rehabilitation medicine, spinal cord injury, stem cell

## Abstract

Human dental pulp stem cells (DPSCs) exhibit neurogenic differentiation potential; however, inflammatory conditions may impair this process. This study investigated the impact of tumor necrosis factor alpha (TNF‐α) on the neurodifferentiation potential of DPSCs and the modulatory role of the pro‐resolving lipid mediators lipoxin A4 (LXA4) and resolvin D1 (RvD1). DPSCs were isolated and subjected to neuronal differentiation for 21 days. Cells were treated with different concentrations of TNF‐α (1, 10, 25, and 100 ng/mL) and with LXA4 or RvD1 (10 and 100 nM). After 21 days, neuronal differentiation was associated with a reduction in the stem cell marker OCT3/4 (53% in the absence of TNF‐α and 48.5% in the presence of TNF‐α) and increased expression of the early neuronal marker doublecortin (87% in the absence of TNF‐α and 116% in the presence of TNF‐α). The intermediate neuronal marker βIII‐tubulin increased by 135% in the absence of TNF‐α but decreased by 40% in its presence. The reduction in βIII‐tubulin induced by 25 ng/mL TNF‐α was partially attenuated by treatment with 100 nM RvD1 (*p* = 0.02) or 10 nM LXA4 (*p* = 0.02). These findings suggest that LXA4 and RvD1 help preserve the neural differentiation potential of DPSCs cultured under inflammatory conditions.

## 1 Introduction

Neuroregenerative stem cell therapy faces several challenges, including the lack of standardized protocols, the selection of cell types that yield the best therapeutic results, limited access to neural stem cells, and the difficulty of cell differentiation in the absence of an inflammatory environment (Carvalho et al. 2024; Khan et al. 2023). Since 2000, dental pulp has been identified as a rich source of neural crest stem cells that are easily obtainable, highly proliferative in culture, and capable of differentiating into neural‐like cells in vitro (Gan et al. 2020). Tooth‐derived stem cells can be obtained from both extracted third molars (human adult dental pulp stem cells [DPSCs]) and from exfoliated deciduous teeth, which allows their harvesting and expansion with relatively limited ethical concerns (Fu et al. 2022; Gan et al. 2020; Jenkner et al. 2024; Kabatas et al. 2018; Luo et al. 2018). They originate from the cranial neural crest and express early markers of both mesenchymal and neuroectodermal stem cells (Luo et al. 2018). DPSCs are pluripotent and can differentiate into osteoblasts, chondrocytes, adipocytes, endothelial cells, and neural‐like cells (Gan et al. 2020; Jenkner et al. 2024; Luo et al. 2018). They express trophic factors that promote neuronal proliferation and survival (Kabatas et al. 2018; Luo et al. 2018).

Transplantation of human DPSCs into the damaged spinal cord has been reported to enhance neuro‐recovery in a rodent model of spinal cord injury (SCI) by inhibiting apoptosis and preserving neural fibers/myelin sheaths, inhibiting axonal growth inhibitors, and differentiating into mature oligodendrocytes (Fu et al. 2024; Kabatas et al. 2018). These neuro‐regenerative properties have not been demonstrated in embryonic stem cells, adult bone marrow stromal cells, or other stem cell populations (Lukomska et al. 2019). Therefore, DPSCs have been investigated as potential candidates for neuroregenerative therapies in conditions such as stroke, traumatic brain injury, and SCI (Fu et al. 2022; Kabatas et al. 2018).

SCI is associated with secondary inflammation, which may limit the in vivo neuronal differentiation of transplanted DPSCs. With disruption of the blood‐spinal cord barrier after SCI, neutrophils infiltrate the damaged cord, triggering inflammatory cytokine production, cell death, and the subsequent release of toxic metabolites, furthering tissue damage (Albashari et al. 2020; Zivkovic et al. 2021). Furthermore, inflammatory scar formation has been shown to inhibit spinal cord regeneration (Albashari et al. 2020; Zivkovic et al. 2021). Little is known about how inflammatory conditions affect the neural differentiation potential of DPSCs. Therefore, we investigated the effect of tumor necrosis factor alpha (TNF‐α) treatment on this process in vitro.

Resolvins and lipoxins are endogenous anti‐inflammatory agents derived from omega‐3 and omega‐6 fatty acids, respectively (Abdelmoaty et al. 2013). These lipid mediators have demonstrated pro‐resolving potential in animal models ranging from asthma to colitis, in addition to promoting regeneration and wound healing, thereby reversing tissue damage (Abdelmoaty et al. 2013; Albuquerque‐Souza et al. 2020; Martini et al. 2016; Park et al. 2023). Intrathecal injection of lipoxin A4 (LXA4) and resolvin D1 (RvD1) has been shown to attenuate inflammatory hypersensitivity in mice (Abdelmoaty et al. 2013; Martini et al. 2016; Park et al. 2023). However, neither mediator has been studied in experimental SCI. We therefore tested the hypothesis that LXA_4_ and RvD1 will mitigate the inflammation‐induced suppression of neural differentiation in DPSCs.

## 2 Materials and Methods

### 2.1 Isolation and Culture of DPSC

DPSCs were isolated from impacted third molars, premolars, or deciduous teeth of seven healthy donors who underwent routine dental care at the ADA Forsyth Institute. All donors provided a signed informed consent or assent form prior to the use of their teeth, as per the study protocol approved by the Forsyth Institutional Review Board (IRB 037). Samples were selected from our tooth biorepository based on the following donor characteristics: absence of medical comorbidities, no active medication use (except for vitamin supplements), and absence of periodontal disease or tooth decay. The extracted pulp was plated and cultured in basal medium, DMEM/F12 supplemented with 15% FBS, 100 U/mL non‐essential amino acids (Gibco Life Technologies), 100 U/mL penicillin and streptomycin, and glutamine (Gibco Life Technologies). Outgrowth of fibroblast‐like cells occurred after 3–4 days, and at that point, the pulp was replated. The outgrowing cells were passaged after reaching 80% confluence, and passage 4 was used for all experiments. Cells were seeded in duplicate at a density of 2 × 10^4^ cells/well. Neuronal differentiation was induced by replacing DMEM/F12 + 15% FBS culture media with neuronal differentiation media (NeuroCult NS‐A Differentiation Kit‐Human, StemCell Technologies Inc., Tukwila, WA, USA) 48 h after seeding. Cells were cultured under neuronal induction conditions for up to 21 days with culture media changed twice a week (Al‐Maswary et al. 2022; Király et al. 2009; Liu et al. 2024).

### 2.2 TNF‐α, LXA4, and RvD1 Treatment

Twenty‐four hours after cell seeding, TNF‐α at concentrations of 1, 10, 25, and 100 ng/mL (PeproTech, Rocky Hill, NJ, USA) was added to cultures of undifferentiated cells. TNF‐α (25 ng/mL; PeproTech, Rocky Hill, NJ, USA), LXA_4_ (10 and 100 nM; Cayman Chemical, Ann Arbor, MI, USA), or RvD_1_ (10 and 100 nM; Cayman Chemical, Ann Arbor, MI, USA) diluted in basal culture medium (DMEM/F12), were added to cultures of both differentiated and undifferentiated cells starting 48 h after seeding.

Varying concentrations of TNF‐α (1, 10, 25, and 100 ng/mL, PeproTech, Rocky Hill, NJ, USA), LXA_4_ (10 and 100 nM, Cayman Chemical, Ann Arbor, MI, USA), or RvD1 (10 and 100 nM, Cayman Chemical, Ann Arbor, MI, USA) were added to the culture of both differentiated and non‐differentiated cells starting 48 h after seeding. In some experiments, cells were treated with both TNF‐α and LXA_4_ or RvD1.

### 2.3 Cellular Metabolic Activity

The metabolic activity of cells was determined using the 3‐(4,5‐dimethylthiazol‐2‐yl)−2,5‐diphenyltetrazolium bromide (MTT) assay at various time points to assess TNF‐α‐induced toxicity in DPSCs. Cells were incubated for 4 h in 5 mg/mL thiazolyl blue tetrazolium bromide (Sigma Aldrich, St. Louis, MO, USA). The precipitate was then suspended in 2‐propanol (Sigma Aldrich, St. Louis, MO, USA), and absorbance was determined in triplicate (Al‐Maswary et al. 2022; Király et al. 2009; Liu et al. 2024).

### 2.4 Real‐Time PCR

Induction of inflammatory cytokine gene expression was determined by real‐time PCR to optimize TNF‐α treatment conditions. Total RNA was extracted from cells using Trizol (Life Technologies). Ten microliters of total RNA was reverse‐transcribed to cDNA using iScript cDNA Synthesis Kit (Bio‐Rad, Hercules, CA, USA). One microliter of cDNA was used as a template for real‐time PCR amplification reactions, performed using the iQ SYBR Green Supermix (Bio‐Rad) in combination with 19 µL of each gene‐specific primer, according to the manufacturer's instructions (Supporting Information S2: Table 1). Glyceraldehyde 3‐phosphate dehydrogenase was used as an endogenous control. PCR reactions were conducted in duplicate using the following conditions: initial denaturation for 3 min at 95°C, followed by 40 cycles of 15 s denaturation at 95°C and 60 s annealing at 60°C. Relative quantification was used for statistical analyses (Al‐Maswary et al. 2022; Király et al. 2009; Liu et al. 2024).

### 2.5 Flow Cytometry

Expression of stem cell and neuronal cell markers was determined by flow cytometry. Cells were fixed with 4% paraformaldehyde for 30 min, permeabilized with 0.1% Triton X‐100 for 10 min and subsequently blocked with TBST in 5% bovine serum albumin (BSA) for 20 min. Cells were incubated for 1 h at room temperature with the following primary antibodies against stem cells markers, rabbit anti‐OCT3/4 (Abcam, 1:200), and neuronal markers, rabbit anti‐doublecortin (Abcam, 1:50) and mouse anti‐β III‐tubulin (Abcam, 1:100). Fluorescence was achieved by incubating with secondary antibodies (Life Tech, Carlsbad, CA, goat anti‐rabbit IgG or goat anti‐mouse IgG, dilution 1:500) for 1 h at room temperature in a dark room. Cells were then subjected to flow cytometry, and the percentage of cells expressing each marker was calculated (Al‐Maswary et al. 2022; Király et al. 2009; Liu et al. 2024).

### 2.6 Immunocytochemistry

Immunocytochemistry was performed on cells according to standard protocols. Briefly, cells were cultured on glass cover slips under varying conditions, washed 3× 10 min in 1X PBS + 0.1% (vol/vol) Tween‐20, fixed in 4% paraformaldehyde (Sigma‐Aldrich, St. Louis, MO, USA) in PBS for 10 min, permeabilized with 0.1% Triton X‐100 (Sigma‐Aldrich, St. Louis, MO, USA) in PBS for 10 min, and washed 3× 10 min in ice‐cold PBS. The cells were then incubated with the following primary antibodies (OCT 1:100, Doublecourtin 1:200; and β‐III Tubulin 1:100, Abcam, Cambridge, MA, USA) in PBS with 3% BSA (Sigma‐Aldrich, St. Louis, MO, USA) for 1 h in a humidified chamber at 4°C. The cells were then washed 3× 10 min in 1X PBS + 0.1% (vol/vol) Tween‐20 and incubated for 1 h at room temperature with the biotinylated secondary antibodies (100 μL/coverslip, diluted 1:250 to 1:750 in blocking buffer, Life Tech, Carlsbad, CA, goat anti‐rabbit IgG or goat anti‐mouse IgG) in a humidified chamber. Finally, cells were washed 3× 5 min in 1X PBS, incubated for 1 min with 1 mg/mL 4′6′‐diamidin‐2‐phenylindol (Vector Laboratories, Burlingame, CA), rinsed in 1X PBS, and the coverslips were mounted with Vectashield mounting medium and sealed. Images were acquired using a Carl Zeiss Axioplan fluorescence microscope (LSM 410, Zeiss, Jena, Germany) (Al‐Maswary et al. 2022; Király et al. 2009; Liu et al. 2024).

### 2.7 Statistical Analysis

Each analysis was performed in duplicate using cells from all donors. For cell metabolic activity and cytokine expression, median and interquartile ranges were calculated for each condition. Mann‐Whitney tests were used; *p* < 0.05 was considered statistically significant. For flow cytometry markers, the mean and standard deviation were considered. One‐way ANOVA, complemented by the Tukey test, was used.

## 3 Results

### 3.1 Donor Characteristics

Donor characteristics at the time of extraction are presented in Table 1. The age range was from 10 to 38 years. The majority was White (57,14%). One donor was an active smoker at the time of extraction. One donor reported vitamin D supplementation. No other medication use was reported. No donor reported an active medical problem at the time of extraction. All extracted teeth were in good condition, without decay, caries, or periodontal disease.

**Table 1:** Donor characteristics.

Study ID	Age	Gender	Race	Active smoker	Medications	Tooth
P1	29	F	White	No	None	Lower 3rd molar (#17)
P5	38	M	Black	Yes	None	Upper 3rd molar (#1)
P6	10	F	White	No	None	Primary upper canine (H)
P8	35	F	Asian	No	Vitamin D supplements	Upper 3rd molar (#1)
P10	14	F	Black	No	None	Upper 1st premolar (#5)
P17	30	F	White	No	None	Lower 3rd molar (#17)
P18	36	M	White	No	None	Upper 3rd molar (#1)

### 3.2 Treatment With TNF‐α Blocks Neuronal Differentiation of DPSC

**Figure 1:**
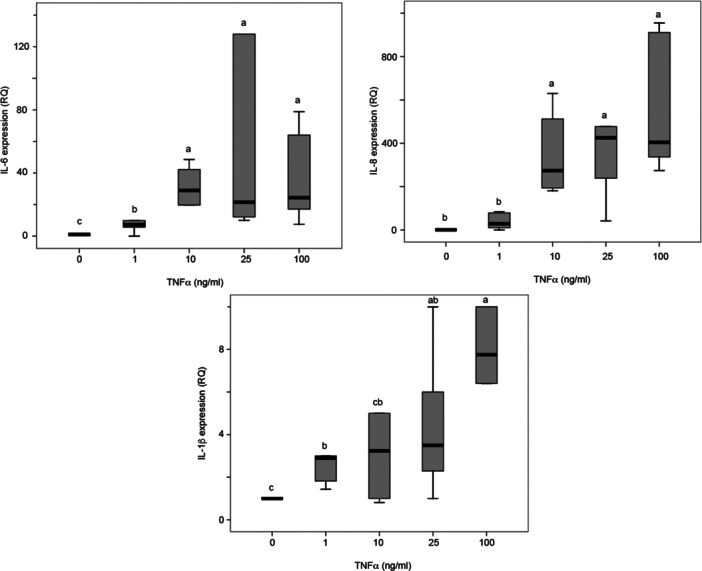
IL‐6, IL‐8, and IL‐1β gene expression (RQ values) by HDPCs submitted to different concentrations of TNF‐α (0, 1, 10, 25, and 100 ng/mL), for 24 h. The box contains 50% of the data points, and the middle line of the box is the median. The tips of the projecting bars show minimum and maximum values. *n* = 7. Mann–Whitney, different letters indicate statistically significant differences (*p* < 0.05).

**Figure 2:**
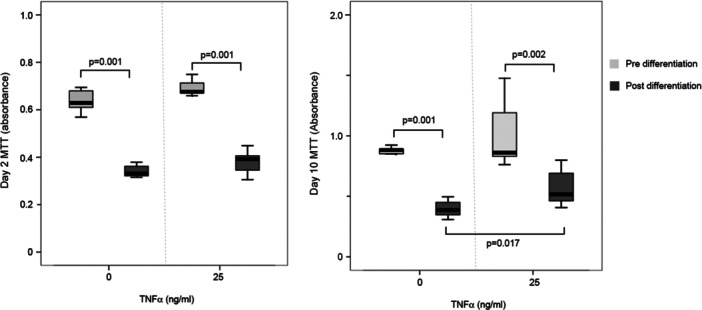
Cell viability detected by MTT Assay (absorbance values) by HDPCs submitted to neuronal differentiation or kept in DMEM/F12 for 2 days or 10 days in the presence (0 ng/mL) or absence (25 ng/mL) of TNF‐α. The box contains 50% of the data points, and the middle line of the box is the median. The tips of the projecting bars show minimum and maximum values, *n* = 7. Mann–Whitney, *p* < 0.05.

TNF‐α treatment of DPSCs was optimized by assessing changes in cytokine production and metabolic activity. All doses of TNF‐α induced significant increases in the production of the inflammatory cytokines IL‐6, IL‐8, and IL‐1β after 24 h of treatment (Figure 1). Decreased metabolic activity was observed at 100 ng/mL TNF‐α (Supporting Information: S1). The concentration of 25 ng/mL TNF‐α was selected for all subsequent experiments because it induced significant inflammation without excessive cytotoxicity to DPSCs. In addition, neural induction resulted in a significant reduction in the metabolic activity of DPSCs at both 2 and 10 days (Figure 2). When only differentiated cells were compared, neural induction in the presence of 25 ng/mL TNF‐α resulted in significantly higher metabolic activity at day 10 compared with cells induced in the absence of TNF‐α (Figure 2, *p* = 0.017).

**Figure 3:**
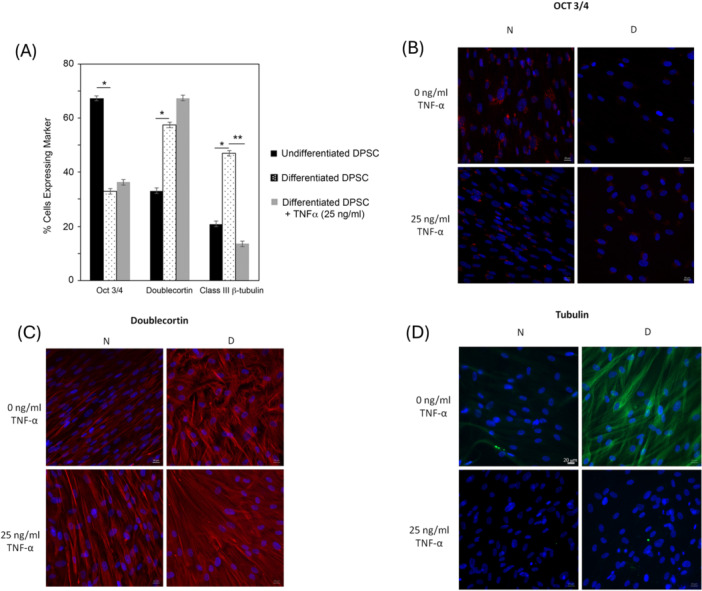
(A) OCT 3/4, Doublecortin and β‐III tubulin production by HDPCs subjected to neuronal differentiation or kept in DMEM/F12 for 21 days in the presence (25 ng/mL) or absence (0 ng/mL) of TNF‐α. Bar graphs indicating the mean values and standard deviation, *n* = 7. One‐way ANOVA complemented by Tukey, *p* < 0.05. (B) OCT 3/4, (C) Doublecortin, and (D) β‐III tubulin immunofluorescence images representative of HDPCs subjected to neuronal differentiation or kept in DMEM/F12 for 21 days in the presence (25 ng/mL) or absence (0 ng/mL) of TNF‐α.

After 21 days of neuronal induction, flow cytometry results demonstrated a significant reduction in the percentage of cells expressing the stem cell marker OCT3/4 (Figure 3A, 53% in the absence of TNF‐α, 48.5% in the presence of TNF‐α). A significantly higher number of stem cells expressed the early neuronal marker doublecortin (Figure 3A, 87% in the absence of TNF‐α, 116% in the presence of TNF‐α). Production of the intermediate neuronal marker β‐III‐tubulin increased by 135% in the absence of TNF‐α and decreased by 40% in the presence of TNF‐α (Figure 3A), demonstrating failure to progress to late neuronal differentiation under inflammatory conditions. These findings were also confirmed by immunocytochemistry (Figure 3B–D for OCT3/4, doublecortin, and β‐III‐tubulin, respectively).

### 3.3 LXA4 and RvD1 Mitigate the Effects of TNF‐α on Neuronal Induction of DPSCs

**Figure 4:**
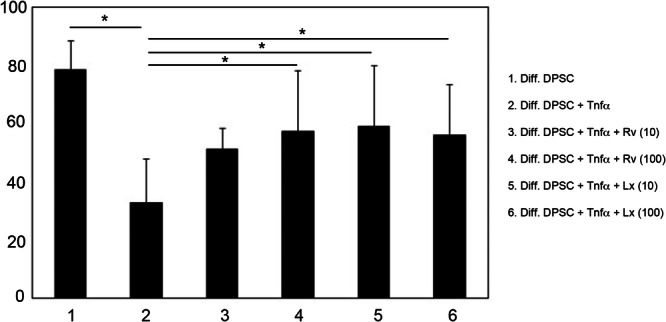
β III‐tubulin production by flow cytometry, after LXA_4_, RvD1, or TNF‐α (25 ng/mL). Bar graphs display the mean values and standard deviations, with *n* = 7. One‐way ANOVA complemented by Tukey's post hoc test, *p* < 0.05. *Statistically significant difference between groups.

We tested the effect of LXA_4_ or RvD1 on mitigating TNF‐α‐induced suppression of DPSC neuronal differentiation. We confirmed that 25 ng/mL TNF‐α significantly reduced β‐III‐tubulin production (Figure 4, 27% decrease, *p* = 0.0002). This reduction was partially blocked by treatment with either RvD1 (100 nM) or LXA_4_ (10 nM). We found a significant 78% increase in β III‐tubulin expression in cells undergoing neural induction in the presence of both TNF‐α and 100 ng/mL RvD1 (32% for 25 ng/mL TNF‐α vs. 57% for 25 ng/mL TNF‐α + RvD1, *p* = 0.02). Similarly, we found a significant 84% increase in β III‐tubulin expression in cells undergoing neural induction in the presence of both TNF‐α and 10 nM LXA_4_ (32% for 25 ng/mL TNF‐α vs. 59% for 25 ng/mL TNF‐α + LXA_4_, *p* = 0.02). Results were similar with LXA_4_ at the 100 nM dose (*p* = 0.03) and approached significance with RvD1 at 10 nM (*p* = 0.09).

## 4 Discussion

The global incidence of SCIs is estimated to be 40 to 80 million cases per year, with more than 18,000 occurring in the United States (Zawadzka et al. 2021). Such injuries are characterized by an initial phase in which damage results directly from the trauma itself, followed by a secondary phase in which damage is caused by local inflammation (Anjum et al. 2020; Bonosi et al. 2022). During inflammation, interleukins and TNF‐α are released, which can trigger neuronal apoptosis, glial scar formation, and axonal demyelination (Anjum et al. 2020; Bonosi et al. 2022). In this study, we evaluated the neural‐inducing capacity of human DPSCs under inflammatory conditions. We also assessed the capacity of endogenous pro‐resolution lipid mediators, LXA_4_ and RvD1, to attenuate TNF‐α‐induced inhibition of neural differentiation in human DPSCs.

As demonstrated by the MTT assay, TNF‐α treatment using 25 ng/mL resulted in increased cellular metabolic activity at 2 and 10 days. Cellular differentiation is generally associated with reduced proliferative activity and lower metabolic demand, whereas undifferentiated cells tend to maintain higher metabolic activity. Thus, the increase in metabolic activity observed in the presence of TNF‐α may be related to the inhibition of neuronal differentiation, allowing DPSCs to remain in a more metabolically active state compared with cells undergoing neural maturation (Cacci et al. 2005; Keohane et al. 2010). However, this increase may also reflect a possible stimulation of cell proliferation induced by the inflammatory environment (Widera et al. 2006). Since the MTT assay does not distinguish between increased metabolic activity and changes in cell proliferation, this finding should be interpreted with caution.

DPSCs have great potential to promote neurorecovery and limit functional deficits after SCI (Gan et al. 2020). Although TNF‐α was not shown to be toxic to DPSCs, neural differentiation of these cells is impaired in the presence of TNF‐α, as demonstrated by immunocytochemistry and flow cytometry at 21 days of induction, when DPSCs presented considerably lower levels of the neuronal intermediate marker βIII‐tubulin. Similarly to our study, it was reported that, in a medium richer in interleukins and TNF‐α, the expression of β‐III‐tubulin by stem cells undergoing differentiation was lower than in a medium poorer in inflammatory factors. This finding corroborates the hypothesis that the inflammatory environment ultimately interferes with the neural differentiation of DPSCs (Jung et al. 2016). In contrast, Bueno et al. found significant levels of β‐III‐tubulin upon neural differentiation of human periodontal ligament stem cells (Bueno et al. 2019). However, they did not simulate an inflammatory environment in their research. Since inflammation is an inherent response after SCI, future therapies using stem cell transplantation should consider the microenvironment of cytokines and inflammatory mediators in which the cells must proliferate and differentiate.

The mechanism by which TNF‐α inhibits stem cell differentiation remains unclear, and various hypotheses have been proposed in the current literature (Sonmez Kaplan et al. 2023). One of them is that TNF‐α, along with other cytokines, activates the transcription factor NF‐κB, which inhibits SOX2 synthesis (Wehling et al. 2009). Studies show that SOX2 is a crucial protein in maintaining pluripotency during the earliest stages of differentiation. Furthermore, SOX2 has been detected in neurogenic regions such as the hippocampus and cerebellum (Pereira and Queiroz 2013). There are no studies directly linking the transcription factor NF‐κB to β‐III‐tubulin; however, given that it is a protein like SOX2, its reduced expression in DPSCs may also be associated with NF‐κB pathway inhibition.

Given that TNF‐α inhibits neural differentiation of DPSCs, it is relevant to discuss strategies that modulate this inflammatory response and, consequently, promote neuroregeneration. Abdelmoaty et al. conducted an in vivo study to investigate whether spinal administration of LXA4 and 17(R)‐RvD1, a more stable analog of RvD1, could reduce TNF‐α and interleukin release, thereby alleviating pain (Abdelmoaty et al. 2013). The authors observed that lipoxins and resolvins inhibit spinal nociceptive processing, suggesting their potential as therapeutic agents.

Complementarily, Albuquerque‐Souza et al. demonstrated that RvE1 and Maresin‐1 promoted periodontal regeneration under inflammatory conditions and also exhibited neuroprotective potential (Albuquerque‐Souza et al. 2020). These authors emphasized that LXA4 may enhance tissue repair by stimulating the release of other lipid mediators, thereby amplifying the reparative response. The findings discussed herein reinforce the notion that lipid mediators such as LXA4 and RvD1 may also modulate inflammation by attenuating TNF‐α signaling (Albuquerque‐Souza et al. 2020).

In the present study, we observed that both LXA4 and RvD1 attenuated the negative effects induced by TNF‐α on DPSC differentiation without exhibiting a dose‐dependent response. This finding may be explained by the fact that both mediators exert their pro‐resolving actions through binding to specific G protein–coupled receptors (GPCRs), particularly ALX/FPR2 and GPR32 (Park et al. 2020; Pirault and Bäck 2018). Due to their high affinity for these receptors, these mediators can trigger effective biological responses even at relatively low concentrations, such as 10 nM.

Although the mechanism by which pro‐resolving lipid mediators mitigate TNF‐α–mediated effects is not yet fully understood, the literature provides hypotheses regarding the possible signaling pathways involved. One possibility is that activation of GPCRs, such as ALX/FPR2 and GPR32, inhibits the NF‐κB pathway, a key signaling axis that controls the transcription of multiple pro‐inflammatory genes, including those involved in TNF‐α synthesis (Isopi et al. 2020; Jaén et al. 2021; Wang et al. 2011). Thus, inhibition of NF‐κB by lipid mediators could explain the observed reduction in the production of inflammatory cytokines (Albashari et al. 2020; Yu et al. 2020).

Pro‐resolving lipid mediators may also modulate other pathways involved in inflammatory signaling, including the JAK/STAT pathway. In this pathway, activation of JAK‐associated receptors promotes phosphorylation of STAT transcription factors, which subsequently regulate gene expression associated with cellular proliferation and differentiation, as well as cytokine synthesis. In the case of LXA4 and RvD1, activation of receptors such as ALX/FPR2 and GPR32 may inhibit the NF‐κB pathway, which is functionally connected to JAK/STAT signaling (Morris et al. 2018; Hu et al. 2021; Philips et al. 2022). Consequently, JAK/STAT signaling may also be less activated, resulting in reduced synthesis of inflammatory cytokines. Although these signaling events were not directly investigated in the present study, future studies evaluating key signaling nodes, such as phosphorylated NF‐κB subunits or neurogenic transcription factors, will be essential to clarify the molecular mechanisms underlying the protective effects of these pro‐resolving mediators.

Therefore, even in the presence of TNF‐α–induced inflammation, human DPSCs maintained neurotrophic factor production and the ability to proliferate. However, the inflammatory environment impaired their neuronal differentiation, preventing progression toward fully mature postmitotic neuronal phenotypes. The pro‐resolving lipid mediators LXA4 and RvD1 attenuated the inhibitory effects of TNF‐α on neuronal marker expression, suggesting that modulation of inflammatory signaling may help preserve the neurogenic potential of DPSCs under inflammatory conditions. Although these findings were obtained in an in vitro model, they highlight the importance of controlling the inflammatory microenvironment during stem cell–based regenerative approaches. Future investigations should also compare acute and chronic inflammatory models to better understand how different inflammatory contexts influence the neurogenic potential of DPSCs. In addition, studies of in vivo models of SCI will be essential to determine whether combining anti‐inflammatory strategies with DPSC‐based therapies may improve neuroregenerative outcomes.

## Author Contributions

**Ana Paula Turrioni:** conceptualization, methodology, validation, formal analysis, investigation, writing – original draft, visualization. **Geovana Pires da Silva:** conceptualization, methodology, investigation, writing – original draft. **Yan Xu:** conceptualization, methodology, investigation, writing – original draft. **Alpdogan Kantarci:** conceptualization, methodology, supervision, writing – review and editing. **Hatice Hasturk:** conceptualization, methodology, supervision, writing – review and editing. **Ricardo A. Battaglino:** conceptualization, methodology, validation, writing – review and editing, visualization, supervision. **Leslie R. Morse:** conceptualization, methodology, validation, formal analysis, writing – review and editing, supervision.

## Ethics Statement

All donors provided a signed informed consent or assent form prior to the use of their teeth, as per the study protocol approved by the Forsyth Institutional Review Board (IRB 037).

## Conflicts of Interest

The authors declare no conflicts of interest.

## Supporting information


**Supplementary Figure:** Cell viability detected by MTT Assay (absorbance values) by HDPCs submitted to neuronal differentiation or kept in DMEM/F12 for 2 days (a) or 10 days (b) in the presence (0 ng/mL) or absence (100 ng/mL) of TNF‐α. The box contains 50% of the data points, and the middle line of the box is the median. The tips of the projecting bars show minimum and maximum values, n = 7. Mann–Whitney, p < .05.

**Supplementary Table:** Primers used in PCR methodology.

## Data Availability

The data that support the findings of this study are available from the corresponding author upon reasonable request.
