# Supplementary figures and images for: Lipoxin A4 and Resolvin D1 Preserve Neural Inductive Capacity of Dental Pulp Stem Cells Cultured Under Inflammatory Conditions
Source: Cell Biol Int. 2026 May 19;50:e70163. doi: 10.1002/cbin.70163 (PMC13184579; doi:10.1002/cbin.70163)

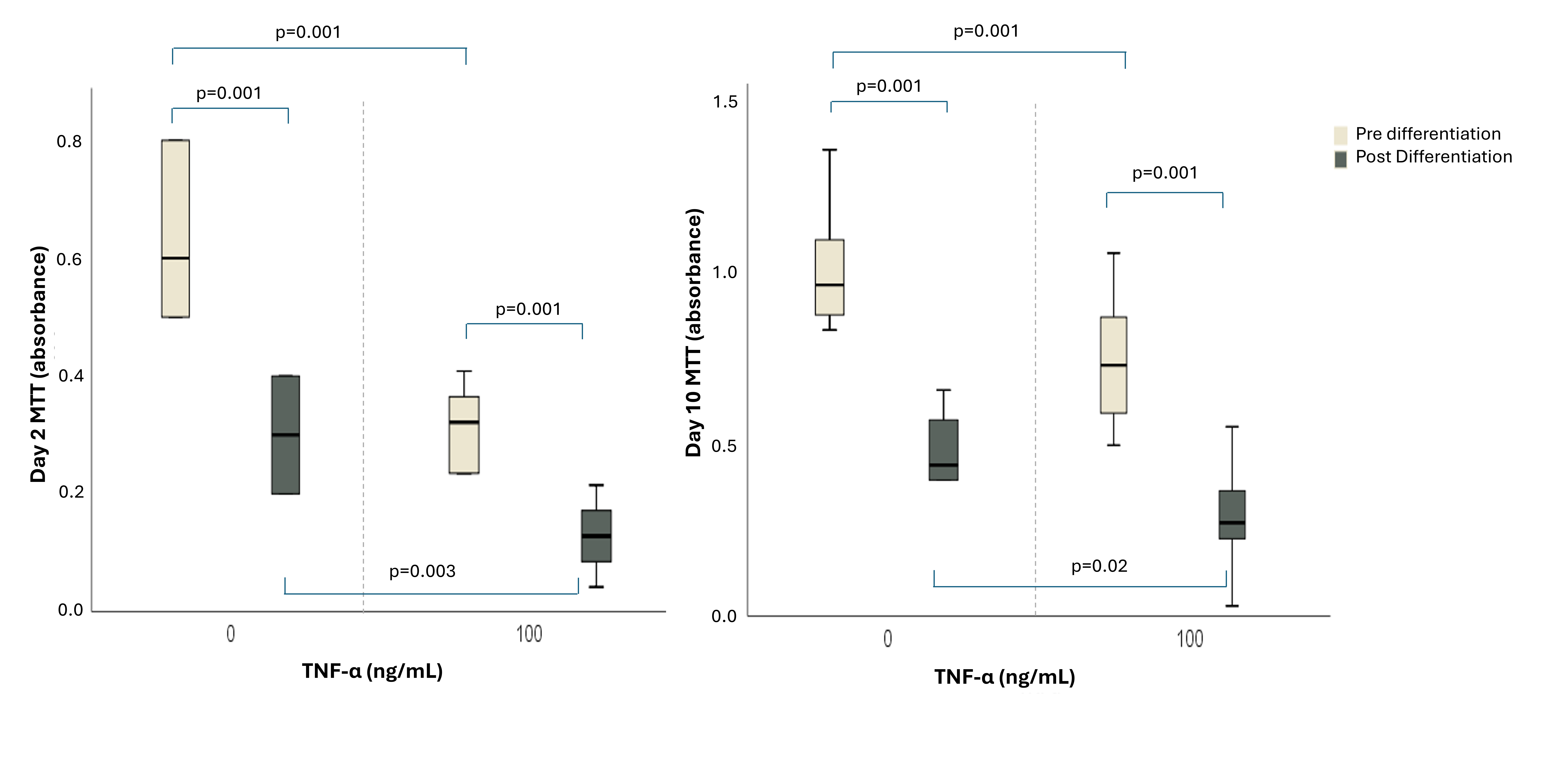

Supplement: Supplementary file 1 — Supplementary Figure: Cell viability detected by MTT Assay (absorbance values) by HDPCs submitted to neuronal differentiation or kept in DMEM/F12 for 2 days (a) or 10 days (b) in the presence (0 ng/mL) or absence (100 ng/mL) of TNF‐α. The box contains 50% of the data points, and the middle line of the box is the median. The tips of the projecting bars show minimum and maximum values, n = 7. Mann–Whitney, p < .05. [file CBIN-50-0-s001.png]
