# Supplementary material for: Lipoxin A4 and Resolvin D1 Preserve Neural Inductive Capacity of Dental Pulp Stem Cells Cultured Under Inflammatory Conditions
Source: Cell Biol Int. 2026 May 19;50:e70163. doi: 10.1002/cbin.70163 (PMC13184579; doi:10.1002/cbin.70163)
Supplement: Supplementary file 2 — Supplementary Table: Primers used in PCR methodology. [file CBIN-50-0-s002.docx]

**Supplementary table:** Primers used in PCR methodology

| **Following primers** | |
| --- | --- |
| IL-6 | Forward: 5’AAATTCGGTACATCCTCGACGG3’  Reverse: 5’GGAAGGTTCAGGTTGTTTTCTGC3’ |
| IL-8 | Forward: 5’ACTGAGAGTGATTGAGAGTGGAC3’  Reverse: 5’AACCCTCTGCACCCAGTTTTC3’ |
| IL-β | Forward: AAGGCGGCCAGGATATAACT-3'  Reverse: 5'- TACGGCCTAAGGCAGGCAGTTG-3' |
| GAPDH | Forward: 5' AGAAAAACCTGCCAAATATGATGAC 3'  Reverse: 5’ TGGGTGTCGCTGTTGAAGTC 3’ |
